# Synthesis, characterization, and biological verification of anti-HER2 indocyanine green–doxorubicin-loaded polyethyleneimine-coated perfluorocarbon double nanoemulsions for targeted photochemotherapy of breast cancer cells

**DOI:** 10.1186/s12951-017-0274-5

**Published:** 2017-05-18

**Authors:** Yu-Hsiang Lee, Yun-Ting Ma

**Affiliations:** 1 Department of Biomedical Sciences and Engineering, National Central University, No. 300, Jhongda Rd., Taoyuan City, 32001 Taiwan, ROC; 2 Department of Chemical and Materials Engineering, National Central University, Taoyuan City, Taiwan, ROC

**Keywords:** Breast cancer, Photothermal therapy, Photodynamic therapy, Chemotherapy, Indocyanine green, Doxorubicin, Perfluorocarbon, Double emulsion

## Abstract

**Background:**

Breast cancer is the most frequently diagnosed cancer and the leading cause of cancer death among females worldwide. Among various types of breast cancer, the human epidermal growth factor receptor 2 (HER2)-overexpressing breast cancer is known to be more aggressive and often resistant to medicinal treatment, leading to an insufficient prognosis and poor susceptibility to chemotherapy and/or hormonal therapy in the current clinic. These circumstances implicate that developing an improved therapeutic strategy rather than persistently changing the anticancer drugs for trying is truly needed to successfully cure this type of breast cancer. In this study, we aimed to fabricate anti-HER2 indocyanine green (ICG)–doxorubicin (DOX)-loaded polyethyleneimine-coated perfluorocarbon double nanoemulsions (HIDPPDNEs) to explore the co-administration of phototherapy and chemotherapy for HER2-overexpressing breast cancer in vitro.

**Results:**

The HIDPPDNE was first characterized as a sphere-like nanoparticle with surface charge of −57.1 ± 5.6 mV and size of 340.6 ± 4.5 nm, whereas the DOX release rates for the nanodroplets within 48 h in 4 and 37 °C were obtained by 8.13 ± 2.46% and 19.88 ± 2.75%, respectively. We then examined the target-ability of the nanostructure and found that the adhesion efficiency of the HIDPPDNEs onto HER2+ MDA-MB-453 cells was threefold higher than the nanodroplets without anti-HER2 antibody, indicating that the HIDPPDNEs are the product with HER2 binding specificity. In comparison to freely dissolved ICG, the HIDPPDNEs conferred an enhanced thermal stability to the entrapped ICG, and were able to provide a comparable hyperthermia effect and markedly increased production of singlet oxygen under near infrared irradiation (808 nm; 6 W/cm^2^). Based on the viability analyses, the results showed that the HIDPPDNEs were effective on cell eradication upon near infrared irradiation (808 nm; 6 W/cm^2^), and the resulting cell mortality was even higher than that caused by using twice amount of encapsulated DOX or ICG alone.

**Conclusions:**

This work demonstrates that the HIDPPDNEs are able to provide improved ICG stability, binding specificity, and enhanced anticancer efficacy as compared to equal dosage of free ICG and/or DOX, showing a high potential for use in HER2 breast cancer therapy with reduced chemotoxicity.

**Electronic supplementary material:**

The online version of this article (doi:10.1186/s12951-017-0274-5) contains supplementary material, which is available to authorized users.

## Background

According to the statistics of the World Health Organization, breast cancer is the most frequently diagnosed cancer and the leading cause of cancer death among females worldwide [1]. Although breast cancer treatments have continuously been advanced over the last decades, metastatic breast cancer remains incurable, and the 5-year overall survival rate is still <25% [2], indicating that an effective therapeutic strategy is still urgently needed. Among various types of breast cancer, the human epidermal growth factor receptor 2 (HER2)-overexpressing breast cancer, which accounts for approximately 30% of breast cancer patients and locates in either primary tumors or metastatic sites, is known to be more aggressive and often resistant to medicinal treatment [3, 4], leading to an insufficient prognosis [5, 6] and poor susceptibility to chemotherapy and/or hormonal therapy [7] in the current clinic, These circumstances implicate that developing an improved therapeutic strategy rather than persistently changing the anticancer drugs for trying is truly needed to successfully cure this type of breast cancer.

Doxorubicin (DOX) is one of the US FDA-approved anticancer drugs and the mechanisms of its antitumor effects have been known to initiate from DNA intercalation and free radical generation [8]. Although DOX has been extensively utilized in a wide spectrum of neoplastic diseases, the dosage of DOX used in the clinical is still highly restricted due to its serious side effects such as bone marrow suppression, dose-dependent cardiotoxicity, liver dysfunction, and increased drug resistance [8, 9]. To circumvent these issues, the co-administration of anticancer agents or therapeutics is often considered a potential regimen for cancer treatment because it may help to decrease the dose of each drug employed and reduce the derived chemotoxicity accordingly, leading to an improved clinical outcome. Among various anticancer therapeutics, noninvasive near infrared (NIR)-based phototherapy has gained increasing attention as an adjuvant to breast cancer chemotherapy because it may provide (1) increased tissue penetration efficacy as compared with that operated by visible light, (2) enhanced membrane permeability for drug uptake, and (3) less toxicity to normal cells/tissues through uses of targeted photosensitive agents and/or spatially controlled light irradiation [10, 11]. Generally, phototherapy is carried out by hyperthermia and/or reactive oxygen species (ROS) generated from the photosensitizers under light illumination in the presence of oxygen; the former may cause thermal ablation of cancer cells [i.e., photothermal therapy (PTT)], while the latter may severely interfere cellular metabolism and thereby trigger programmed cell death [i.e., photodynamic therapy (PDT)] [11–13]. No matter which mechanism is employed, the photosensitizer plays a key role in the effect of phototherapy.

Indocyanine green (ICG) is a US FDA-approved tricarbocyanine dye that can be absorbed and fluoresce in the region of 650–850 nm. Thus far, in addition to serving as a fluorophore agent for use in diagnostic purpose such as NIR image-guided oncologic surgery [14] and fluorescence angiography [15], ICG has been extensively exploited for cancerous phototherapy that includes breast, brain, and skin tumors [16–18] due to its capabilities of heating and singlet oxygen generation upon NIR exposure. However, the drawbacks of ICG such as high aqueous degradability [19] and rapid plasma clearance [20] severely hinder its applicability in the clinic.

Nanomedicine may offer a feasible means for the co-administration of anticancer drugs including ICG and DOX without aforementioned drawbacks because it may provide enhanced bioavailability, improved stability, and security to the payloads [21]. In this study, we sought to fabricate a type of anti-HER2 ICG-DOX-loaded polyethyleneimine (PEI)-coated perfluorocarbon (PFC) double nanoemulsions (HIDPPDNEs) to explore the potential of co-administration of photo- and chemotherapy for HER2-overexpressing breast cancer cells. PFC, a fluorine-substituted derivative of hydrocarbons, is a well-known oxygen transporter since it can dissolve large respiratory gases as compared to water [22], implicating that it is greatly advantageous for use in PDT. PEI was employed as a polycationic interface used for the electrostatic assembling of antibody. Overall we anticipate that the developed HIDPPDNEs are able to (1) potentially protect the entrapped ICG from degradation caused by external stimuli such as light, heat, and/or pH [19, 23], (2) specifically hit the therapeutic region to reduce any off-target cytotoxicity generated from the DOX, and (3) provide an effective cancer treatment with reduced chemotoxicity since the multiplex photochemotherapy may diminish the efficacious dosage of anticancer drug used in the chemotherapy alone. In this paper, we first introduced the fabrication of HIDPPDNEs in detail, followed by the stepwise investigations of their physicochemical properties, functionalities, and anticancer efficacy.

## Methods

### Carboxylation of pluronic F68 copolymer

The introduction of carboxylic acid group onto the end of poly(ethylene oxide)-block-poly(propylene oxide)-block-poly(ethy1ene oxide) (PEO-PPO-PEO block copolymer; Pluronic F68) was conducted by reaction with succinic anhydride as reported previously [24]. Briefly, 6 g of pluronic F68 and 0.2 g of succinic anhydride were first mixed with 4-dimethylaminopyridine (DMAP; 160 mM) and triethylamine (TEA; 140 mM) in a total 10 mL of 1,4-dioxane for 18 h. After removing the 1,4-dioxane by vacuum evaporation, the residue was dissolved in chloroform and the synthetic carboxyl-terminated pluronic F68 (CT-PF68) was precipitated by adding excessive diethyl ether. The unreacted succinic anhydride, DMAP and TEA were removed by using vacuum filtration. After repeating the diethyl ether extraction and filtration for three times, the collected product was vacuum-dried and stored at 4 °C until use. The characteristics of the synthetic CT-PF68 were identified using proton nuclear magnetic resonance (^1^H NMR) technique.

### Fabrication of HIDPPDNEs

**Fig. 1:**
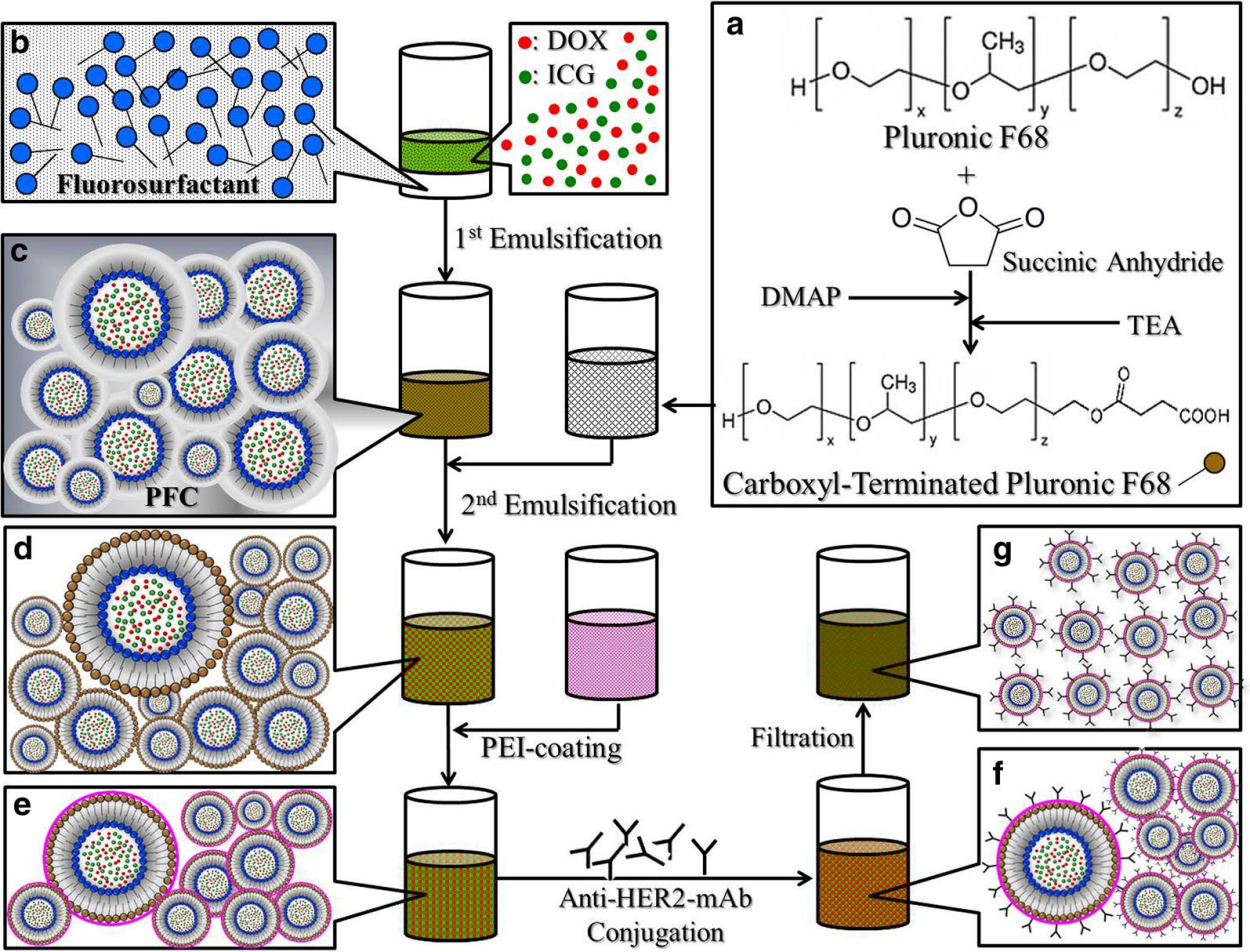
Schematic diagram of the fabrication procedures of the HIDPPDNEs. The CT-PF68 was first synthesized through reaction with succinic anhydride in the presence of DMAP and TEA (**a**). The IDPDNEs structured with fluorosurfactant and CT-PF68 were then formed by using a modified emulsification approach (**b**–**d**), followed by PEI coating (**e**) and anti-HER2-mAb conjugation (**f**) on the nanodroplet surface in sequence to obtain the HIDPPDNEs. To remove excess/unreacted chemicals and simultaneously reduce the size dispersity of the products, the yielded HIDPPDNEs with a broad size range were filtrated through a 0.45-μm filter and exhibited improved size uniformity afterward (**g**)

The ICG-DOX-loaded PFC double nanoemulsions (IDPDNEs) were first prepared by using a modified emulsification approach. Briefly, 6 mg of polyethoxylated fluorosurfactant was first dissolved in 1.2 g of perfluorooctyl bromide (PFOB). Next, 550 μL of methanol (50% v/v) containing 1-mg ICG and 1-mg DOX was added to the surfactant-PFOB solution. The mixture was then sonicated under ice bath to obtain the primary water-in-PFC (W1/PFC) emulsion. Afterward the primary emulsion was added slowly into PBS containing CT-PF68 (5% w/w) followed by sonication to obtain the W1/PFC/W2 double emulsions (i.e., IDPDNEs). After washed twice with PBS, the IDPDNEs were mixed with PEI and incubated at room temperature for 60 min to obtain the ICG-DOX-loaded PEI-coated PFC double nanoemulsions (IDPPDNEs). After washed twice with PBS, the IDPPDNEs were mixed with 100 μg of anti-HER2-mAb and incubated at room temperature for 2 h to obtain the final product of HIDPPDNEs. The HIDPPDNEs were then washed, resuspended in PBS, and lyophilized. The overall procedures of the HIDPPDNE fabrication are illustrated in Fig. 1.

### Evaluation of effectiveness of antibody conjugation

The presence and bioactivity of anti-HER2-mAbs on the nanodroplet surface were verified by using the fluorescent anti-mouse immunoglobulin G (IgG) secondary antibody (F-IgG-SAb) as the probe. The fluorescence level expressed from the F-IgG-SAb-treated nanodroplets was detected using fluorescent microscopy and spectrofluorometry performed with 488 and 525 nm of excitation and emission wavelength, respectively. In this study, the intensity of fluorescence was quantitatively represented by relative fluorescence units (RFUs), and the fluorescence levels in all types of nanodroplet were analyzed using the normalized RFUs against the background signal.

### Evaluation of the physicochemical properties of HIDPPDNEs

The size distribution and surface charge of the HIDPPDNEs were measured using the dynamic light scattering (DLS) technique. The morphology of the HIDPPDNEs was detected using a scanning electron microscope with 20 kV accelerating voltage. The encapsulation efficiency (*E*) of the drug (ICG or DOX) was calculated by the formula:

1 $$E = \frac{{W_{t} - W_{s} }}{{W_{t} }} \times 100\%$$

where *W*_t_ is the total amount of ICG or DOX used for the HIDPPDNE manufacture and *W*_s_ denotes the amount of unencapsulated drug molecules remaining in the supernatant. Both *W*_t_ and *W*_s_ were determined by UV–Vis spectrometry (λ_abs_ = 780 nm for ICG; 485 nm for DOX) according to Beer-Lambert’s law. The loading rate of the payload (ICG or DOX) in the HIDPPDNEs (*R*; wt%) was evaluated by the formula:

2 $$R = \frac{W}{{W_{NE} }} \times 100\%$$

where *W*_NE_ is weight of the HIDPPDNEs and *W* denotes the weight of the ICG or DOX encapsulated in the HIDPPDNEs examined (~*W*_t_ × *E*).

### Analysis of HIDPPDNE stability

To evaluate the size variation and stability of the anti-HER2-mAb on the nanodroplet surface under biological environment, the HIDPPDNEs with defined amount were added into human serum albumin (HSA)-containing PBS solution (0.5 mM) and the mixtures were incubated at 37 °C for 0, 0.5, 2, 6, 12, and 24 h, respectively. At each time point, the HIDPPDNEs were washed twice with PBS and then subjected to DLS and aforementioned F-IgG-SAb-mediated spectrofluorometry for size measurement and antibody quantification, respectively. A standard curve of HIDPPDNE concentration (% v/v) vs. intensity of fluorescence (RFU) resulted from F-IgG-SAb conjugation was established prior to the experiment. The fluorescence intensity obtained from the IDPPDNEs with the same treatment was employed as the control. The fluorescence levels in all nanodroplet settings were analyzed using the normalized RFUs against the background signal.

The thermal stability of the HIDPPDNE-entrapped ICG and the release kinetics of the entrapped DOX at 4 and 37 °C were measured in this study. All the HIDPPDNE samples were wrapped in foil to prevent light illumination during the experiment. After treatment for 2, 4, 24, and 48 h, the HIDPPDNEs and their supernatant collected by centrifugation were subjected to spectrophotometry at λ_abs_ = 780 nm (for the HIDPPDNEs) and 485 nm (for the supernatant) to analyze the amount of ICG remaining in the nanodroplets and the amount of DOX released to the bulk phase, respectively. The cumulative release rate of DOX (*CR*_D_) at each time point was calculated using the formula:

3 $$CR_{D} = \frac{{M_{Dt} }}{{M_{D0}^{{}} }} \times 100\%$$

where *M*_D0_ represents the amount of DOX originally loaded in the HIDPPDNEs tested and *M*_Dt_ denotes the amount of DOX detected in the supernatant at a specific time *t* > 0. The degradation rate coefficient (*k*_d_) of ICG in each group was determined based on the dynamic method [25]:

4 $$\frac{{C_{t} }}{{C_{0} }} = \exp ( - k_{d} \times t)$$

where *C*_0_ and *C*_t_ denote the concentrations of ICG in the HIDPPDNEs or PBS (control) at time *t* = 0 and a specific time *t* > 0, respectively.

### Cell culture

The MDA-MB-453 cells (HER2(+) human breast metastatic carcinoma cell line; ATCC, Rockville, MD) were cultured in Leibovitz’s L-15 medium supplemented with 10% fetal bovine serum (FBS), 2-mM l-glutamine, and 100-U/mL penicillin–streptomycin, and maintained at 37 °C without CO_2_. Canis macrophages (DH82/CRL-10389; ATCC) were cultivated in minimum essential medium supplemented with 15% FBS, 0.1-mM non-essential amino acids, 2-mM l-glutamine, 1-mM sodium pyruvate, and 100-U/mL penicillin–streptomycin, and maintained in a 37 °C incubator balanced with 5% CO_2_ and 100% humidity.

### Examination of macrophage uptake efficiency of HIDPPDNEs

The macrophage uptake efficiency of the HIDPPDNEs in vitro was examined by analyzing the percentage of non-engulfed nanodroplet after contacted with the macrophages for 24 h. Briefly, 6 × 10^6^ DH82 cells were aliquoted into 12 wells of a 24-well culture plate and incubated at 37 °C for 24 h. Afterward a defined amount of HIDPPDNEs and IDPPDNEs were separately added to six wells and co-cultured with the cells at 37 °C for 0, 0.5, 2, 6, 12, and 24 h, respectively. At each time point, the cells in both groups were washed twice with PBS and detected by fluorescence microscopy. In addition, the proportion of the HIDPPDNE which was not engulfed by DH82 cells; the remaining rate of HIDPPDNE (*R*), was calculated by comparing the nanoparticle number presenting in the collected supernatant (*N*_t_; *t* > 0) to the amount provided to the cells in the beginning (*N*_0_; *R* = *N*_t_/*N*_0_). In this study, the amount of HIDPPDNE in the medium (*N*) was estimated using the formula:

5 $$N = \frac{\% PFC \times V}{{V_{DE} }}$$

where %PFC denotes the percentage (v/v) of PFOB in the HIDPPDNE sample that was obtained through gravimetric measurement and regression analysis as reported previously [26]. *V* represents the total volume of the sample and *V*_*DE*_ is the theoretical volume of a single HIDPPDNE determined based on the result of DLS measurement.

### Examination of binding specificity of HIDPPDNEs

The target specificity of the HIDPPDNEs was determined by examining the adsorption efficiency of the HIDPPDNEs in the HER2-expressing breast cancer cells with and without competitive molecules. Briefly, 3 × 10^6^ MDA-MB-453 cells were aliquoted into six wells of a 24-well culture plate and incubated at 37 °C for 24 h. For the non-competitive assay, the HIDPPDNEs and IDPDNEs with equal ICG/DOX content were separately added to one of the six wells and incubated at 37 °C for 4 h. In terms of the HER2 competitive assay, the HIDPPDNEs were added to the other three wells and co-cultured with the cells in the presence of 0.5, 1, or 2 μg/mL of free anti-HER2-mAb at 37 °C for 4 h. The group without a nanodroplet was employed as the control. After wash twice with PBS, the cells were detected by fluorescence microscopy and the intensities of both ICG- and DOX-derived fluorescence were measured using spectrofluorometry performed with excitation/emission wavelength of 750/838 and 485/590 nm, respectively. In this study, the cellular uptake efficiency of the HIDPPDNEs was analyzed using the normalized RFUs against the control.

### Measurement of HIDPPDNE-induced hyperthermia effect

To evaluate the photothermal effect of the HIDPPDNEs, 200-μL PBS containing HIDPPDNEs with defined ICG equivalent concentrations were separately irradiated by an 808-nm laser with an intensity of 6 W/cm^2^ in one well of a 96-well culture plate. The temperature of each group was recorded every 30 s for 5 min using a digital thermometer.

### Measurement of production of HIDPPDNE-induced singlet oxygen

The productions of singlet oxygen generated from the HIDPPDNEs with and without preoxygenated treatment under 808-nm laser exposure with an intensity of 6 W/cm^2^ were measured using the singlet oxygen sensor green (SOSG) kit (Life Technologies, Carlsbad, CA, USA) according to the manufacturer’s instructions. The oxygenation was performed by injecting 100% oxygen into the nanodroplet medium for 15 min before use. The level of SOSG-induced fluorescence in each group was measured by spectrofluorometry every 60 s for 5 min and was quantitatively represented by RFUs.

### In vitro cytotoxicity assay

To evaluate the photochemotherapeutic capacity of the HIDPPDNEs, 6.4 mL of culture medium containing 3.2 × 10^6^ MDA-MB-453 cells was aliquoted into 32 wells of a 96-well culture plate and incubated at 37 °C for 24 h. Afterward, the freely dissolved ICG and DOX was added to ten and five wells, respectively, the HIDPPDNEs were added to ten wells, and the HIDPPDNEs with preoxygenated treatment were added to five wells. The concentrations of free ICG and DOX were corresponding to the doses provided by the HIDPPDNEs, and were set as 0.5, 1, 2, 4, and 10 μM for ICG and 0.25, 0.5, 1, 2, and 5 μM for DOX. After incubation at 37 °C for 4 h, the cells in the ten wells with free ICG, five wells with HIDPPDNEs, five wells with preoxygenated HIDPPDNEs, and one well without agents were washed twice with PBS followed by treatment of NIR irradiation (808 nm; 6 W/cm^2^) for 5 min. The cells in the five wells that originally treated with free ICG were then maintained with DOX and the concentration of DOX in each well was determined based on the aforementioned ICG-DOX dosage collocation. The cells in the other 16 wells were maintained at 37 °C with normal growth medium. Cells in all groups were incubated at 37 °C for an additional 24 h followed by viability analyses. For the groups without NIR treatment, the viabilities of the cells in the five wells with free DOX, five wells with HIDPPDNEs, and one well without agents were directly measured after 24-h incubation. The cell viability in each well was evaluated using both hemocytometry and calcein-AM/propidium iodide (concentration ratio = 2:3) staining assay.

### Statistical analysis

*A*ll data were acquired from three independent experiments and are presented as the mean ± standard deviation (s.d.). Statistical analyses were conducted using MedCalc software in which comparisons for one condition between two groups were performed by Student’s *t* test with a significance level of *P* < 0.05 throughout the study.

## Results and discussion

### Analysis of carboxylated Pluronic F68

**Fig. 2:**
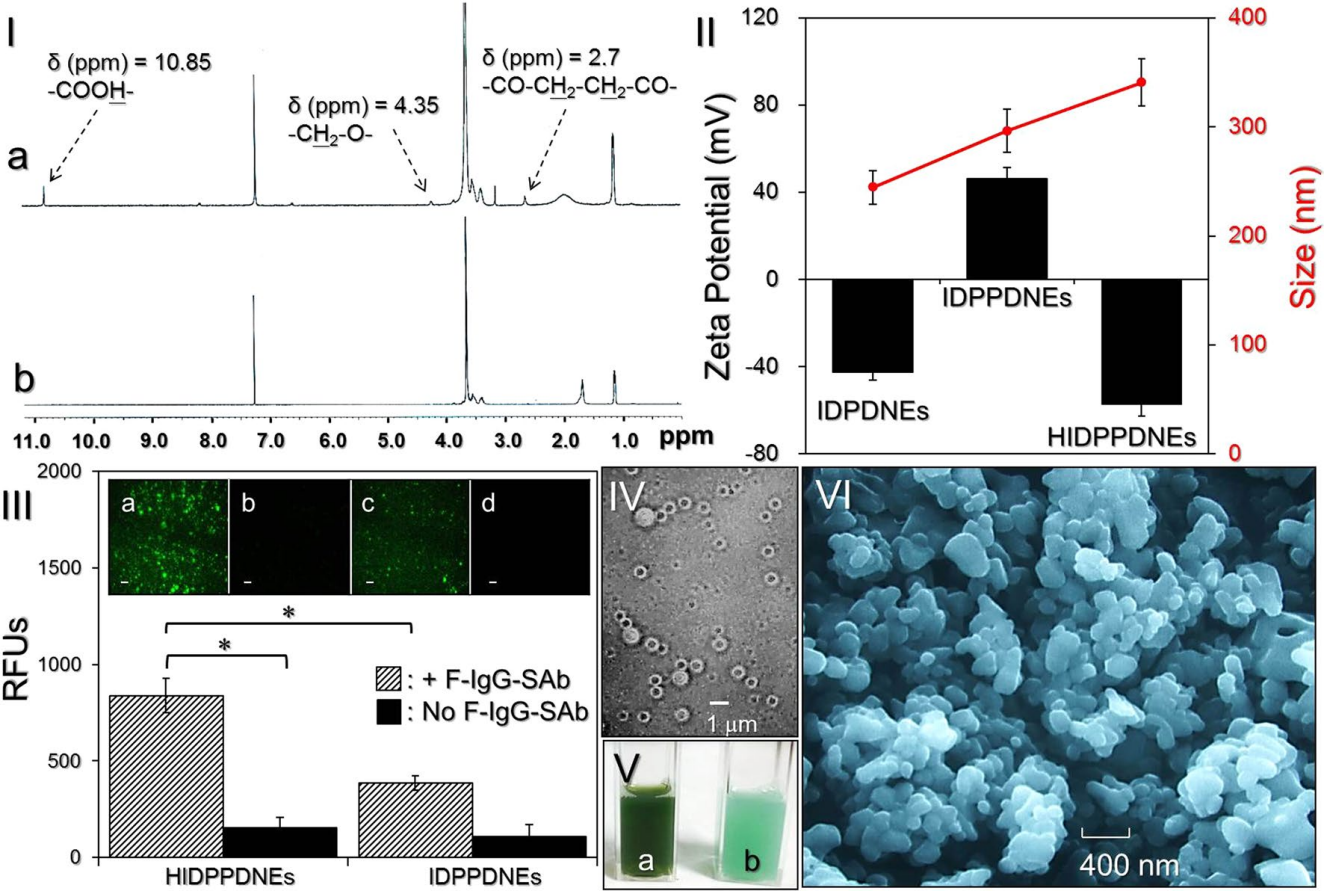
Assessment of physicochemical properties of the HIDPPDNEs. **I** The ^1^H NMR spectra of the synthetic CT-PF68 (*a*) and Pluronic F68 (*b*). **II** Size (*red line*) and surface charge (*black bars*) of the IDPDNEs, IDPPDNEs, and HIDPPDNEs. **III** Verification of the presence and bioactivity of anti-HER2-mAbs on the HIDPPDNE surface. The inset photographs are the representative fluoromicroscopic images of HIDPPDNEs (*a*, *b*) and IDPPDNEs (*c, d*) with (*a, c*) and without (*b, d*) F-IgG-SAb conjugation at ×200 magnification. *Scale bar* 10 μm. The intensity of fluorescence expressed from each group was measured using spectrofluorometry at 488/525 nm of excitation/emission wavelength and was quantitatively represented by RFUs. Values are mean ± s.d. (n = 3). *P < 0.05. **IV** Photomicrographic image of HIDPPDNEs before filtration at magnification of ×400. **V** Appearance of the HIDPPDNE (*a*) and ICG-loaded PEI-coated PFC double nanoemulsion (*b*) solutions. **VI** SEM image of the HIDPPDNEs at magnification of ×10,000

The success of Pluronic F68 carboxylation was verified by analyzing the characteristic peaks of the Pluronic F68 with and without interaction with succinic anhydride using ^1^H NMR spectroscopy. In the spectrum of the succinic anhydride activated Pluronic F68 (i.e., CT–PF68, Fig. 2I-a), the peak at δ (ppm) = 2.7 denotes the –CO–CH_2_–CH_2_–CO– protons contributed from the succinic anhydride group; the peak at δ (ppm) = 4.35 represents the –CH_2_–O– protons attributed from the ethylene oxide group adjacent to the succinic anhydride group, and the peak at δ (ppm) = 10.85 denotes the carboxylic group (–COOH–) in the structure of the product. All the aforementioned peaks are absent in the spectrum of Pluronic F68 (Fig. 2I-b), demonstrating that the synthesis of CT-PF68 through the succinic anhydride activation was successfully carried out.

### Verification of HER2 antibody on the nanodroplet surface

Figure 2II shows the size and zeta potential of fabricated IDPDNEs, IDPPDNEs, and HIDPPDNEs. The IDPDNEs exhibited size of 245 ± 3.6 nm and negative surface charge of −42.6 ± 3.6 mV because plethora of carboxylic moieties were distributed on the emulsion surface. After coated with PEI and anti-HER2 monoclonal antibody (mAb) in sequence, the nanodroplets continuously enlarged and exhibited opposite electrical property for the IDPPDNEs and HIDPPDNEs. These outcomes indicate the success of PEI and anti-HER2-mAb decoration on the nanodroplet surface. The size of the HIDPPDNE was 340.6 ± 4.5 nm with a polydispersity index of 0.08–0.14 after filtration, and the surface charge was approximately −57.1 ± 5.6 mV based on the DLS measurement. The representative graphs of size distribution and zeta potential for IDPDNEs, IDPPDNEs, and HIDPPDNEs were shown in Additional file 1: Figures S1, S2, respectively.

We further examined the bioactivity of the conjugated antibody using the secondary antibody assay. As shown in Fig. 2III, the HIDPPDNEs displayed remarkable fluorescence expression after treatment with the F-IgG-SAb (Fig. 2III, inset image a) and the level of fluorescence was 5.5-fold (*P* < 0.05) higher than that obtained from the HIDPPDNEs without F-IgG-SAb (Fig. 2III, inset image b). Furthermore, the fluorescence level of the HIDPPDNEs with F-IgG-SAb was approximately 2.2-fold (*P* < 0.05) higher than that obtained from the F-IgG-SAb-treated IDPPDNEs (Fig. 2III, inset image c), showing that the enhanced fluorescence expression in the former group was mainly contributed from the first-secondary antibody conjugation instead of F-IgG-SAb-PEI electrostatic adsorption. These results clearly show that the HIDPPDNEs have an affinity to the corresponding secondary antibody, indicating that the anti-HER2-mAbs were certainly bound on the surface of the HIDPPDNEs and were able to provide intact bioconjugation activity after the electrostatic assembling process.

### Characterization of HIDPPDNEs

Figure 2IV exhibits the photomicrographic image of the HIDPPDNEs before filtration whereby the double-layer structure of the nanodroplets is observed. Unlike showing the light green color for the double nanoemulsions with ICG alone (Fig. 2V, b), the green-to-brown emulsified appearance of the HIDPPDNEs (Fig. 2V, a) clearly illustrates the presence of ICG and DOX in the nanodroplets. Furthermore, according to the SEM image (Fig. 2VI), it can be seen that the produced HIDPPDNEs remained intact particulate shape without collapse after the fabrication procedures including high-speed centrifugation, agitation, and filtration. Based on the Eq. (1), the encapsulation efficiencies of ICG and DOX in the HIDPPDNEs are 91.6 ± 2.1% and 60.7 ± 8.1%, respectively, while the loading rates of ICG and DOX in the HIDPPDNEs are approximately 0.71 ± 0.12 and 0.2 ± 0.03 wt%, respectively, calculated based on the Eq. (2).

### Stability of the HIDPPDNEs under protein-enriched environment

Biological fluid is principally comprised of proteins and a number of biomolecules including amino acids and ions, and such environment may influence the hydrodynamic behavior and/or rigidity of nanostructure. Although nonspecific antibody adsorption while still keeping the nanoparticles in negative charge (e.g., HIDPPDNEs) may provide them stability in colloidal solution [27], the electrostatic interactions may be shielded in biological fluid and thus the stable nanostructure may be destabilized due to adsorption of external molecules/proteins and/or desorption of antibodies on the particle surface [28]. These circumstances may adversely affect the functionality of the HIDPPDNEs in vivo and diminish their applicability in the clinic accordingly.

**Fig. 3:**
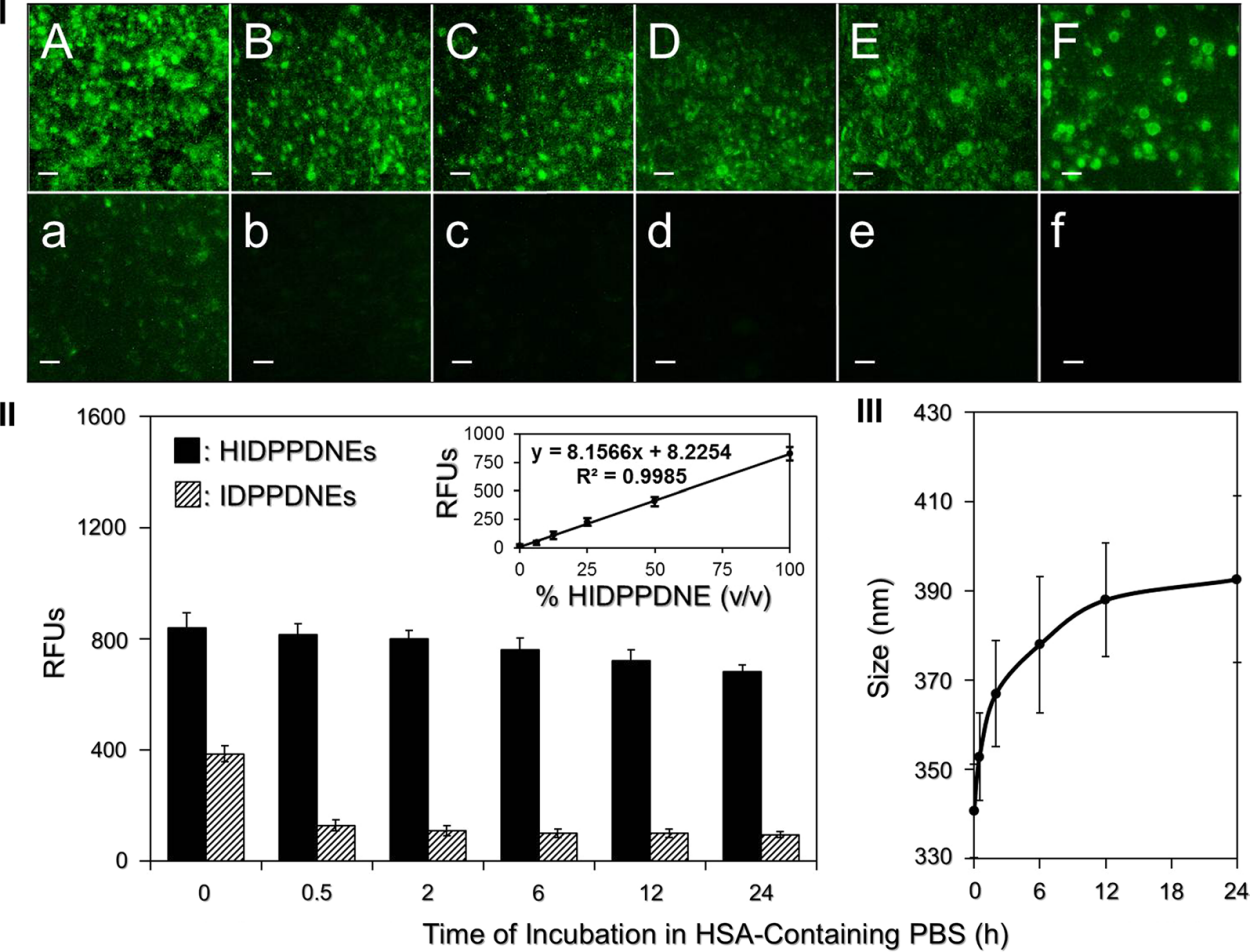
Assessment of stability of the HIDPPDNEs under protein-enriched environment. **I** Representative fluoromicroscopic images of F-IgG-SAb-bound HIDPPDNEs (*A*–*F*) and IDPPDNEs (*a–f*) which were pre-incubated in a 37 °C HSA buffer for 0 (*A*/*a*), 0.5 (*B*/*b*), 2 (*C*/*c*), 6 (*D*/*d*), 12 (*E*/*e*), and 24 (*F*/*f*) h prior to conjugation with the fluorescent probe. All images were photographed at ×200 magnification. *Scale bar* 10 μm. **II** Quantitative analyses of the fluorescence levels expressed from the HIDPPDNEs and IDPPDNEs after maintained in a 37 °C HSA buffer for different time. The intensities of the fluorescence were measured using spectrofluorometry at 488/525 nm of excitation/emission wavelength and were quantitatively represented by RFUs. The inset curve presents the correlation between the concentration of the HIDPPDNE (% v/v) and the RFUs expressed that was determined by using spectrofluorometry at 488/525 nm of excitation/emission wavelength. Values are mean ± s.d. (n = 3). **III** Quantitative analyses of the size variation of the HIDPPDNE after maintenance in a 37 °C HSA buffer for different time. Values are mean ± s.d. (n = 3)

To assess the availability of the HIDPPDNE under biological environment, the affinity of the anti-HER2-mAb on the nanodroplet surface in the presence of serum proteins was examined. As shown in Fig. 3, the RFUs of the F-IgG-SAb-conjugated HIDPPDNEs slightly decreased ~20% after pre-incubation with HSA buffer at 37 °C for 24 h (Fig. 3I, A–F). Based on the linear correlation between quantity of anti-HER2-mAb and RFUs expressed (Fig. 3II, inset curve), this result implicates that about 80% of anti-HER2-mAbs were still able to be retained on the nanodroplet surface after 24-h HSA treatment. To ensure that the fluorescence was resulted from the F-IgG-SAb-anti-HER2-mAb association instead of conjugation with serum proteins, we further implemented the same experiment by using the IDPPDNEs and found that the RFU remarkably reduced threefolds after mixed with the HSA for 30 min (Fig. 3I, a–b), and was maintained at a similar level in the rest of the time for 24 h (Fig. 3I, b–f). Since the surface of the IDPPDNE will certainly be attached by HSA due to electrostatic interaction before addition of the fluorescent probes, our data clearly show that the F-IgG-SAb cannot conjugate with the HSA and all the fluorescence expressed from the group of HIDPPDNE (Fig. 3I, A–F) was attributed to the conjugation of two antibodies. These results suggested, that the targetability of the HIDPPDNE in the presence of serum proteins was still applicable. In addition to the availability of surface antibody, the dimension of the HIDPPDNE was concurrently monitored during the 24-h incubation with HSA in 37 °C. As plotted in Fig. 3III, it can be observed that the nanodroplet size exponentially rose in the first 12 h and then slowly increased afterward, resulting in a size increase of 15% after 24 h.

### Macrophage internalization of the HIDPPDNEs in vitro

Opsonization occurs when foreign objects are covered with proteins that function in directing phagocytes toward them. Generally, the net charge and/or hydrophobic character of a particle (e.g., HIDPPDNE) may cause undesirable interaction with opsonin proteins and trigger phagocytosis of macrophages afterward, leading to an immediate particle removal from the body [29]. In this study, with confirmed stability of the HIDPPDNEs under a protein-enriched environment, we sought to investigate the anti-phagocyticity of the HIDPPDNE under impact of macrophage clearance.

**Fig. 4:**
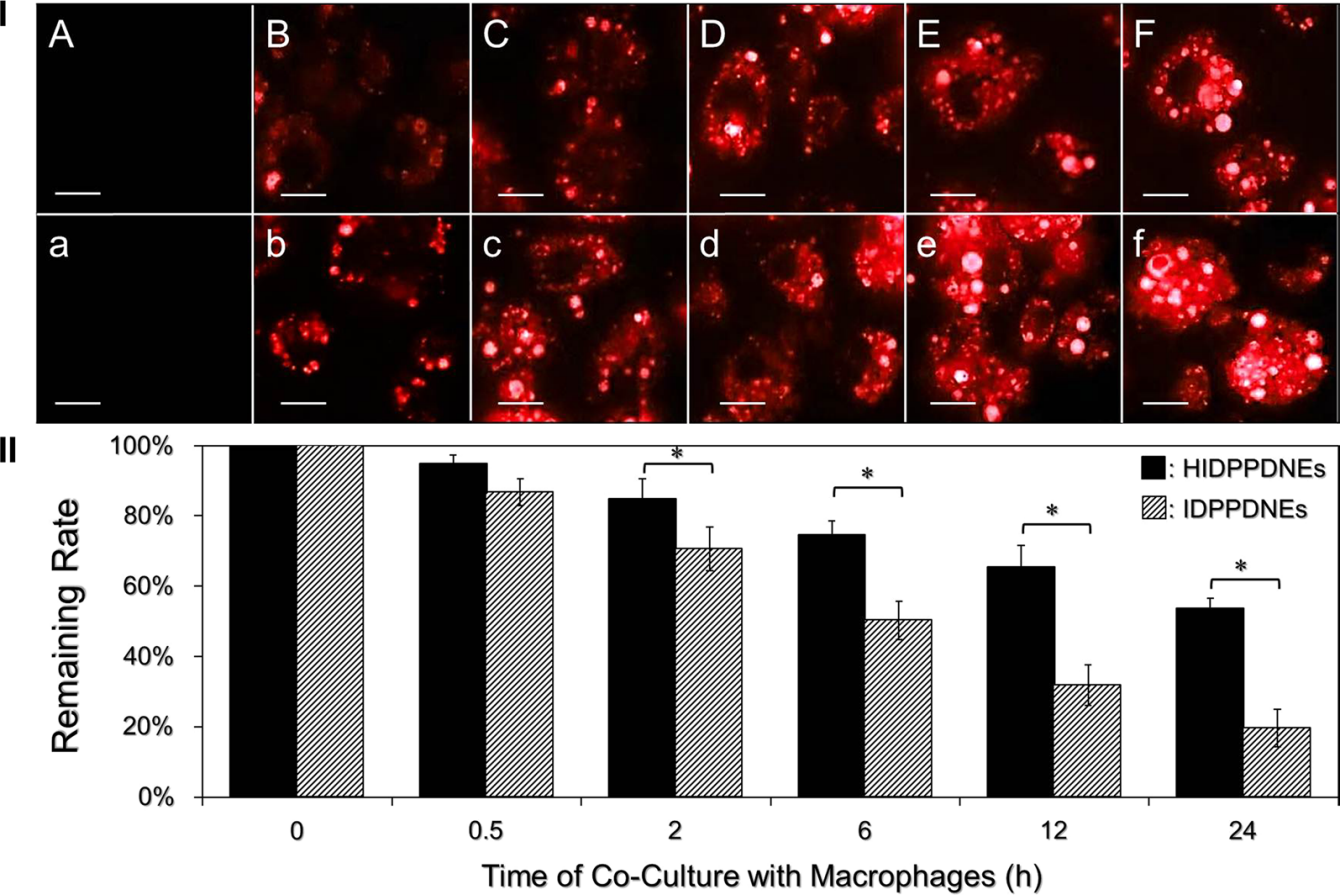
Evaluation of macrophage uptake efficiency of the HIDPPDNEs in vitro. **I** Representative DOX-derived fluoromicroscopic images of DH82 cells after co-culture with the HIDPPDNEs (*A–F*) or IDPPDNEs (*a*–*f*) at 37 °C for 0 (*A*/*a*), 0.5 (*B*/*b*), 2 (*C*/*c*), 6 (*D*/*d*), 12 (*E*/*e*), and 24 (*F*/*f*) h. The cells in all groups were washed twice with PBS prior to the photographing. All images were photographed at ×400 magnification. *Scale bar* 15 μm. **II** Quantitative analyses of the remaining rates of HIDPPDNEs or IDPPDNEs after co-culture with the DH82 cells for different time. Values are mean ± s.d. (n = 3). *P < 0.05

Figure 4 shows the outcomes of nanodroplet internalization performed by DH82 cells under different time for up to 24 h. Through the detections of DOX-derived fluorescence in the cells, it can be observed that the DH82 cells exhibited relatively mild phagocytosis to the HIDPPDNE (Fig. 4I, A–F) as compared with IDPPDNE (Fig. 4I, a–f). We reason that the less efficacy of macrophage internalization for HIDPPDNEs was attributed to their negatively charged surface because anionic particles displayed less electrostatic interaction with cell membrane than cationic ones (i.e., IDPPDNEs) and thus rendered a lower cellular uptake efficiency as reported previously [30]. Furthermore, based on the analysis of particle number before and after treatment with the macrophages using Eq. (5), the results show that >50% of the HIDPPDNEs were able to be kept in free suspension after co-culture with the DH82 cells for 24 h, suggesting that the HIDPPDNEs are highly applicable for use in vivo.

### Thermal stability of HIDPPDNE-entrapped ICG and efficiency of DOX release

**Fig. 5:**
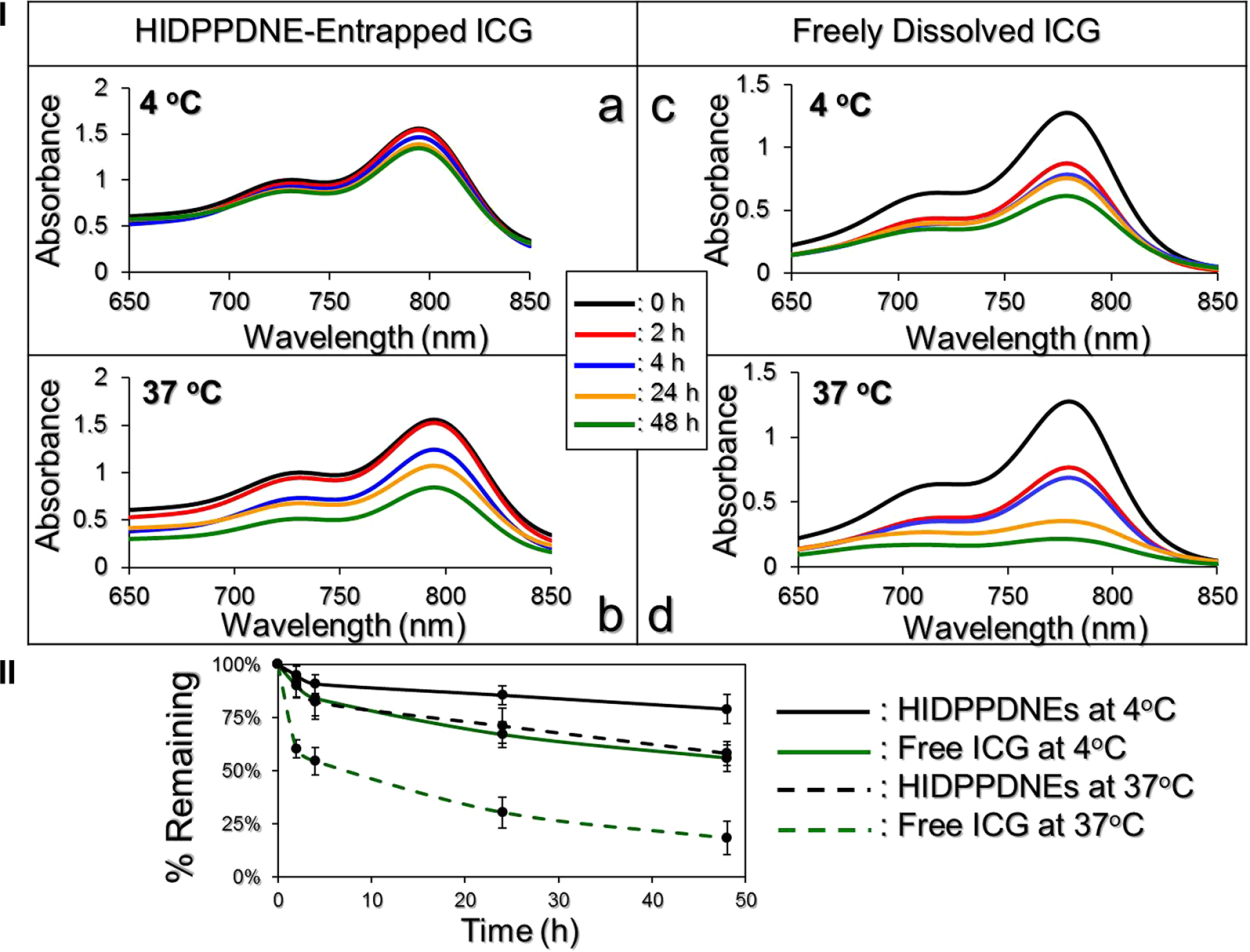
Assessment of the thermal stability of HIDPPDNE-entrapped ICG and freely dissolved ICG in PBS. **I** *a*–*d* show the UV–Vis spectra of HIDPPDNEs (*a, b*) and freely dissolved ICG (*c, d*) in PBS under 4 (*a, c*) or 37 °C (*b, d*) incubation without light illumination for 0, 2, 4, 24, and 48 h. The absorbance at λ = 780 nm in each spectrum denotes the level of ICG remaining in the sample after the treatment. **II** The variation curves of ICG remaining in the HIDPPDNEs or in the PBS under 4 and 37 °C incubation for 48 h. Values are mean ± s.d. (n = 3)

Figure 5I presents the degradation profiles of the HIDPPDNE-entrapped ICG (Fig. 5I, a, b) and freely dissolved ICG in PBS (Fig. 5I, c, d) under incubation at 4 or 37 °C in the dark for 48 h. After analyzing the absorbance values at λ = 780 nm for each set (Fig. 5II), we found that the percentages of ICG remaining in the emulsion matrix were 1.5- and 3.2-fold higher than those remaining in the PBS after 48 h at 4 and 37 °C, respectively. Moreover, based on the *K*_d_ analyses (Table 1), the degradability of the HIDPPDNE-entrapped ICG at 4 and 37 °C significantly reduced 2.5 (*P* < 0.05)- and 3.1 (*P* < 0.05)-fold, respectively, as compared to the free ICG with equal treatment for 48 h. These outcomes clearly show that the thermal stability of ICG was markedly improved after entrapped in the HIDPPDNEs.

**Table 1:** Analyses of degradation percentages and degradation rate coefficients for the HIDPPDNE-entrapped ICG and free ICG

Group	% ICG degradation	*k*_d_ (h^−1^)
HIDPPDNE-entrapped ICG		
4 °C in the dark	21.05%^a^	0.0049^a^
37 °C in the dark	39.84%^a^	0.0113^a^
Freely dissolved ICG in PBS		
4 °C in the dark	44.21%	0.0121
37 °C in the dark	81.63%	0.0353

^a^ *P* < 0.05 as compared to the group with freely dissolved ICG under the same heating treatment

**Fig. 6:**
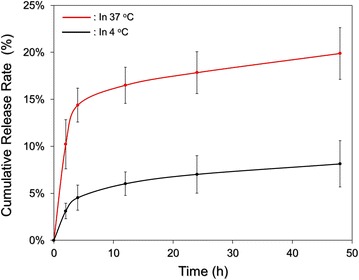
Kinetic release profiles of DOX from the HIDPPDNEs in vitro. The cumulative release curves of the HIDPPDNE-entrapped DOX under 4 and 37 °C in PBS were established by measuring the concentrations of DOX in the supernatant through UV–Vis spectrometry (λ = 485 nm) after treatment for 0, 2, 4, 12, 24, and 48 h. Values are mean ± s.d. (n = 3)

The cumulative percentages of DOX released from the HIDPPDNEs at 4 and 37 °C were concurrently measured, and the results are shown in Fig. 6. Both groups exhibit a two-phased DOX release profile, and the overall release rates after 48-h maintenance in 4 and 37 °C are 8.13 ± 2.46 and 19.88 ± 2.75%, respectively. Such a biphasic drug release profile, consisting of an initial burst release followed by a sustained slow release for the encapsulated DOX, is in agreement with the results reported in a plethora of studies [31, 32]. We reason that the increased burst release of the entrapped DOX in the first few minutes of heating at 37 °C was due to demulsification (i.e., coalescence, Ostwald ripening, and phase inversion/separation) of HIDPPDNE since temperature elevation may accelerate the rate of particle collision due to reduction of emulsion viscosity and increased Brownian motion of the droplets with <2 μm in diameter [33], leading to droplet breakdown and rapid DOX release accordingly.

The fluctuation of emulsion configuration may subsequently reach an equilibrium state and thereby retarded the drug release rate. Moreover, in comparison to the similar products reported previously, the DOX release efficiency of the HIDPPDNEs is lower than that obtained from the DOX-loaded PLGA nanoparticles [32] and/or ICG-DOX-loaded lipid-PLGA nanoparticles [34], implicating that the DOX is relatively stable in the HIDPPDNEs. We speculate that the reduced drug release rete from the HIDPPDNEs was attributed by (1) a higher degree of steric hindrance on the emulsion surface caused by tangled PEI and/or antibody molecules and (2) less reactivity of the nanodroplets because the outward PEI may generate steric and/or electrostatic repulsion [35] to diminish interactions with foreign molecules that conferred an enhanced shelf stability to the HIDPPDNEs in the aqueous medium.

### Binding specificity of HIDPPDNEs

**Fig. 7:**
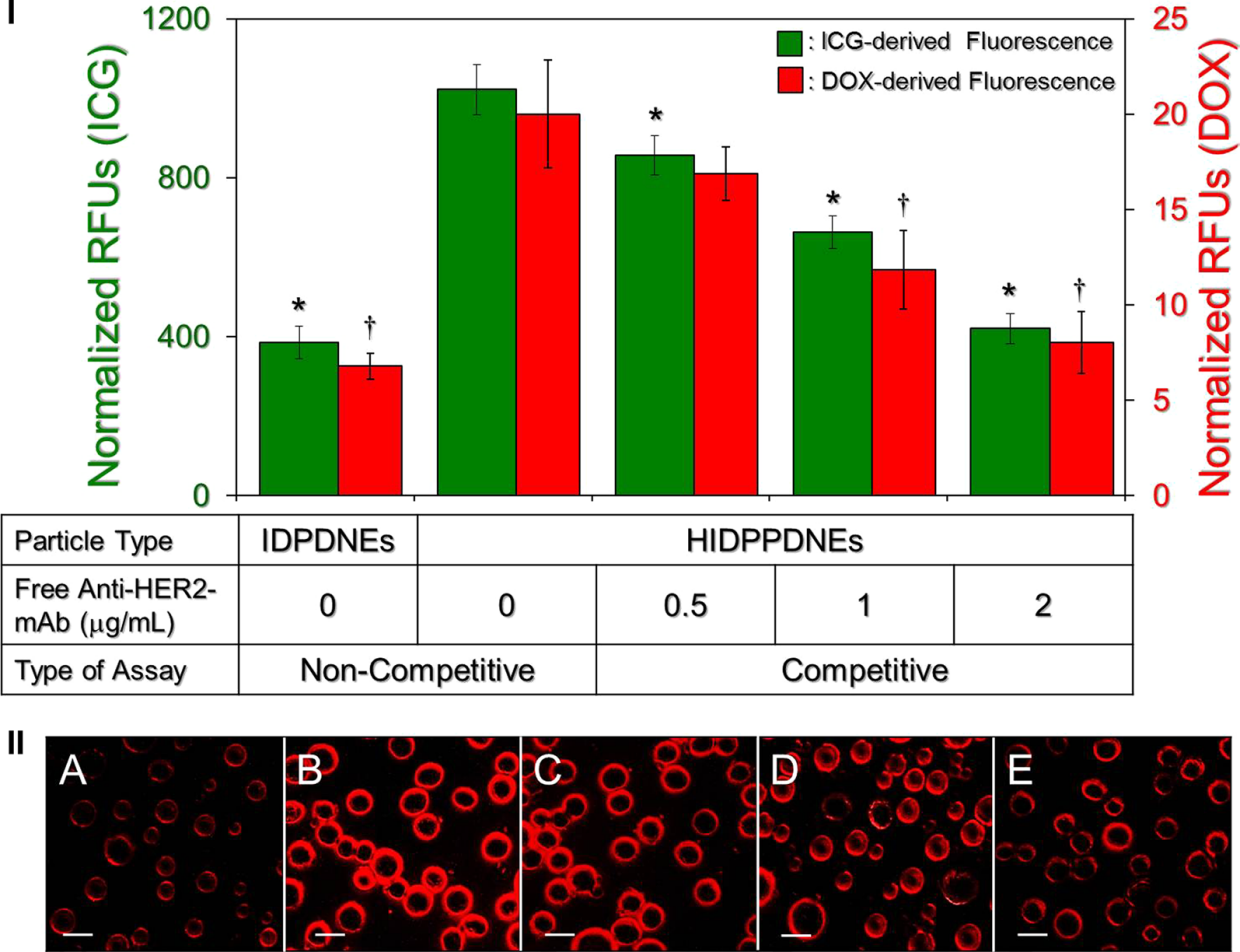
Verification of HER2-binding specificity of the HIDPPDNEs. **I** The *upper panel* shows the analyses of the fluorescence levels expressed from the MDA-MB-453 cells after treated with the IDPDNEs and/or HIDPPDNEs with and without free anti-HER2-mAb. Both types of nanodroplets were used in 1-µM ICG/0.5-µM DOX equivalent concentration to the cells. For the non-competitive assay, cells were treated with IDPDNEs or HIDPPDNEs in the absence of free anti-HER2-mAb for 4 h. For the competitive assay, cells were separately treated with the HIDPPDNEs in the presence of 0.5, 1, or 2 μg/mL of free anti-HER2-mAb at 37 °C for 4 h. Both ICG- and DOX-derived fluorescence were simultaneously detected right after the nanodroplets were removed. The intensities of the fluorescence were measured using spectrofluorometry performed with excitation/emission wavelength of 750/838 and 485/590 nm for detection of ICG and DOX, respectively, and were quantitatively represented by RFUs. Values are mean ± s.d. (n = 3). *P < 0.05 as compared to the ICG-derived RFUs obtained from the HIDPPDNE-treated group without free anti-HER2-mAb. ^†^ *P* < 0.05 as compared to the DOX-derived RFUs obtained from the HIDPPDNE-treated group without free anti-HER2-mAb. **II** The bottom panel shows the DOX-derived fluoromicrographic images of MDA-MB-453 cells after co-cultured with the IDPDNEs (*A*) or HIDPPDNEs (*B*–*E*) in the absence (*A, B*) and presence of 0.5- (*C*), 1- (*D*), or 2-(*E*) μg/mL free anti-HER2-mAb for 4 h. All images were photographed at ×200 magnification. *Scale bar* 10 μm

Figure 7I shows the levels of ICG- and DOX-induced fluorescence expressed from the MDA-MB-453 cells after treatment with IDPDNEs or HIDPPDNEs with and without antibody competition for 4 h. In terms of the non-competitive study, the ICG- and DOX-derived fluorescence levels detected from the HIDPPDNE-treated cells were 2.7 (*P* < 0.05)- and 3 (*P* < 0.05)-fold, respectively, higher than that obtained from the IDPDNE-treated cells. These outcomes are consistent with the fluoromicroscopic observation (Fig. 7II) where the density of the HIDPPDNEs on the MDA-MB-453 cell membrane (Fig. 7II, B) is markedly higher than that performed with IDPDNEs (Fig. 7II, A). To ensure that the increased adhesion of HIDPPDNEs on HER2-positive cells was attributed to the conjugation with cellular HER2 receptors, the binding efficiencies of HIDPPDNE on the MDA-MB-453 cells in the presence of competitive anti-HER2-mAb were further examined. As presented in Fig. 7I, the results show that the ICG-derived RFU significantly decreased ~60% (*P* < 0.05) when the dose of free anti-HER2-mAb was increased from 0 to 2 μg/mL. Similar results can be obtained from detection of the DOX-derived fluorescence using spectrofluorometry (Fig. 7I) and fluorescent microscopy (Fig. 7II, B–E). In terms of the issue that how the two types of nanodroplets exhibited different binding rates, we reason that the uptake of HIDPPDNEs in the HER2-positive MDA-MB-453 cells was conducted by receptor (HER2)-mediated endocytosis, while the mechanism of IDPDNE internalization was performed through adsorptive endocytosis; an efficient means for cancer cells to engulf negatively charged nanoparticles as reported previously [36]. Since receptor-mediated endocytosis is more efficient and specific than adsorptive endocytosis [37], it is predictable that the HIDPPDNEs can be more efficiently internalized/adsorbed by the MDA-MB-453 cells and exhibited markedly increased RFUs as compared to the group with IDPDNEs.

### Effects of hyperthermia and singlet oxygen generation of HIDPPDNEs

**Fig. 8:**
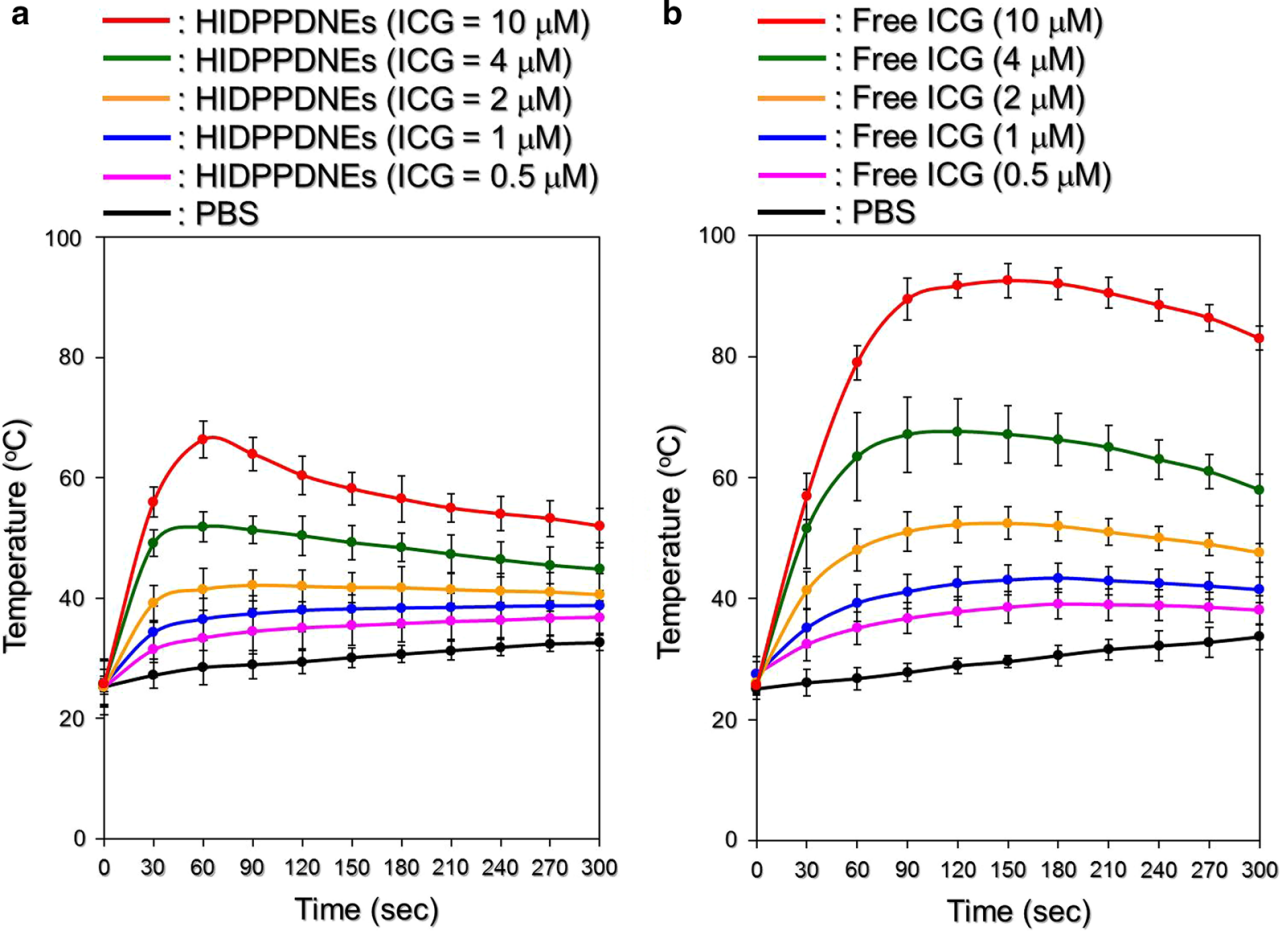
Hyperthermia effects of the HIDPPDNEs under NIR laser irradiation. Upon exposure to an 808-nm laser with an intensity of 6 W/cm^2^, the variations of temperature in the samples of HIDPPDNEs (**a**) and freely dissolved ICG (**b**) with concentrations of 0- (PBS only), 0.5-, 1-, 2-, 4-, and 10-μM ICG equivalent were measured using a digital thermometer every 30 s for 5 min. Values are mean ± s.d. (n = 3)

Figure 8 exhibits the results of the hyperthermia effect generated from the various concentrations of HIDPPDNEs (Fig. 8a) and freely dissolved ICG (Fig. 8b) under 808-nm laser irradiation with an intensity of 6 W/cm^2^ for 5 min. Similar with the free ICG, the temperature in each HIDPPDNE group rapidly increased within the first minute of NIR irradiation and sustained at approximately the same level (groups with ≤2 μM ICG) or slowly declined (groups with >2 μM ICG) thereafter, yielding an increase of 7.3, 11.6, 13, 15.3, 20.4, and 28.2 °C after 5 min of NIR exposure for the HIDPPDNEs with 0- (PBS only), 0.5-, 1-, 2-, 4-, and 10-μM ICG equivalent concentration, respectively. However, one may notice that the level of HIDPPDNE-induced temperature elevation was lower than that obtained from the freely dissolved ICG under the same treatment. Our explanations are as follows: in contrast to the ICG solution that all the ICG molecules simultaneously reacted upon NIR laser irradiation, the hyperthermia effect generated from the HIDPPDNEs was contributed by partially released ICG; therefore, the magnitude of the temperature elevation was relatively moderate compared to that performed by free ICG. Nonetheless, these results show that the HIDPPDNEs are certainly able to provide a dose-dependent hyperthermia effect upon NIR laser irradiation.

**Fig. 9:**
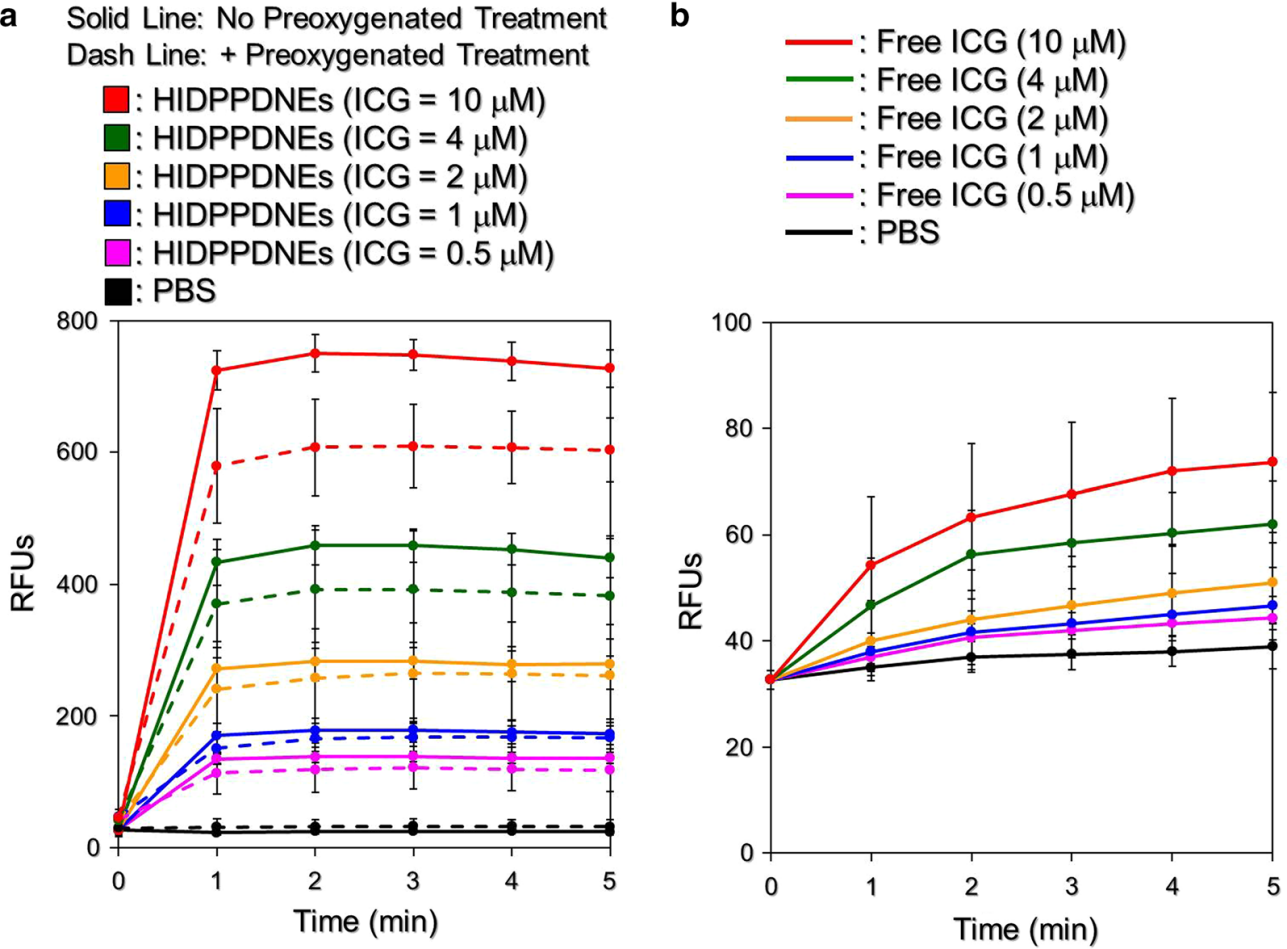
Productions of the HIDPPDNE-induced singlet oxygen under NIR laser irradiation. Upon exposure to an 808-nm laser with an intensity of 6 W/cm^2^, the productions of singlet oxygen generated from the (**a**) HIDPPDNEs with and without preoxygenated treatment and (**b**) freely dissolved ICG in PBS with concentrations of 0- (PBS only), 0.5-, 1-, 2-, 4-, and 10-μM ICG equivalent were measured every 60 s for 5 min. The quantities of singlet oxygen were analyzed based on the intensities of the SOSG-induced fluorescence measured by using spectrofluorometry with excitation/emission wavelength of 488/525 nm. Values are mean ± s.d. (n = 3)

Figure 9 shows the effects of singlet oxygen generation produced by various concentrations of the HIDPPDNEs with and without preoxygenated treatment (Fig. 9a) and freely dissolved ICG (Fig. 9b) within 5-min NIR irradiation. Our data show that the HIDPPDNEs enabled a dose-dependent production of singlet oxygen as performed by free ICG, but the yielded level was enormously higher than that obtained from the same concentration of free ICG. Based on the RFU analysis, the HIDPPDNEs were able to provide 10-fold (*P* < 0.05) more singlet oxygen than the free ICG when the dose of ICG was increased to 10 μM. In addition, it can be seen that the RFUs in the preoxygenated HIDPPDNEs was slightly less than the values obtained from the HIDPPDNEs without preoxygenation, indicating that hyperoxia environment was conversely detrimental to the generation of singlet oxygen. This outcome is consistent with the previous studies [38, 39] and we reason that such overoxygen-mediated quench of singlet oxygen is predominantly caused by oxygen in excited singlet state as reported previously [40]. Nevertheless, these results demonstrate that the HIDPPDNEs are able to provide an increased production of singlet oxygen as compared to the same concentration of free ICG upon NIR laser irradiation, and such enhanced efficacy was attributed to the presence of PFC in the HIDPPDNEs.

The temperature level plays the key role in the effect of PTT. According to previous studies, irreversible cell damage can be obtained after heating at 40–45 °C for 30–60 min [41], while only 4–6 min is sufficient at 50–52 °C [42]. At temperature of >60 °C, the time required to cause irreversible cell damage dramatically decreases because the denaturation of cytoplasmic proteins and/or enzymes may occur shortly and lead to immediate necrosis accordingly [43]. Although a higher temperature may provide more opportunities to eradicate cancer cells, a moderate temperature in the range of 41–43 °C is more often used in the clinic to minimize any possible heat-induced detrimental effect on the surrounding normal tissues/cells [44]. Based on the results shown in Figs. 8 and 9, we reason that the HIDPPDNEs with ≥2-μM ICG equivalent concentration are able to provide both photothermal (*T* ≥ 41 °C) and photodynamic effects for eradication of cancer cells, whereas the phototherapeutic efficacy of HIDPPDNEs with ≤1-µM ICG equivalent concentration is mainly dependent on the photodynamic effect under NIR laser irradiation (808 nm; 6 W/cm^2^).

### Efficiency of DOX release under NIR laser irradiation

**Fig. 10:**
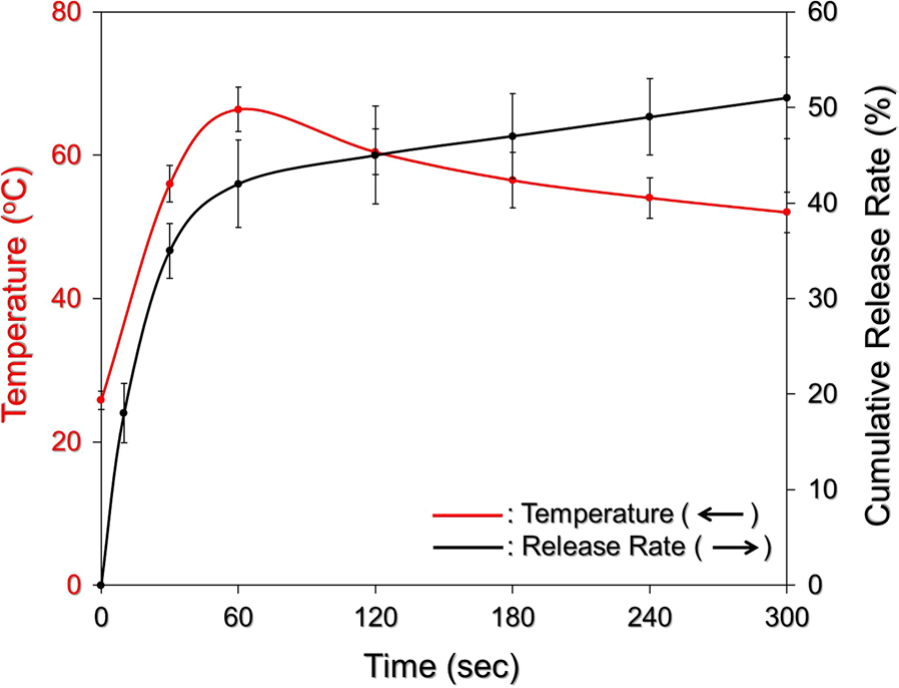
Kinetic release profile of DOX from the HIDPPDNEs under NIR laser irradiation in vitro. The cumulative release curve of the HIDPPDNE-entrapped DOX (*black curve*) under NIR laser irradiation was established by measuring the concentrations of DOX in the supernatant through UV–Vis spectrometry (λ = 485 nm) after irradiated for 0, 10, 30, 60, 120, 180, 240, and 300 s. The NIR exposure was performed using an 808-nm laser with an intensity of 6 W/cm^2^. The red curve indicates the temperature of the HIDPPDNE medium at each time point while the concentration of released DOX was detected. Values are mean ± s.d. (n = 3)

We subsequently examined the efficiency of DOX release under NIR laser irradiation using HIDPPDNEs with 10-μM ICG/5-μM DOX equivalent concentration. As shown in Fig. 10, a two-phased DOX release profile was obtained as it was performed in the absence of NIR exposure (Fig. 6), and the cumulative release rate after 5-min operation was 51.8 ± 4.3%. Given that the temperature of the nanodroplet medium was able to reach ~70 °C within 60-s NIR irradiation which is higher than the melting temperature of the Pluronic F68 (*T*_m_ = ~54 °C) [45], it is reasonable to conclude that the disintegration of the HIDPPDNE was triggered shortly after the NIR irradiation and thereby led to a quick release of DOX in the first minute of NIR treatment (Fig. 10). In comparison with the outcomes shown in Fig. 6, these results clearly show that the efficiency of DOX release from the HIDPPDNEs can be facilitated by NIR laser treatment (808 nm; 6 W/cm^2^).

### In vitro cytotoxicity of HIDPPDNEs

**Fig. 11:**
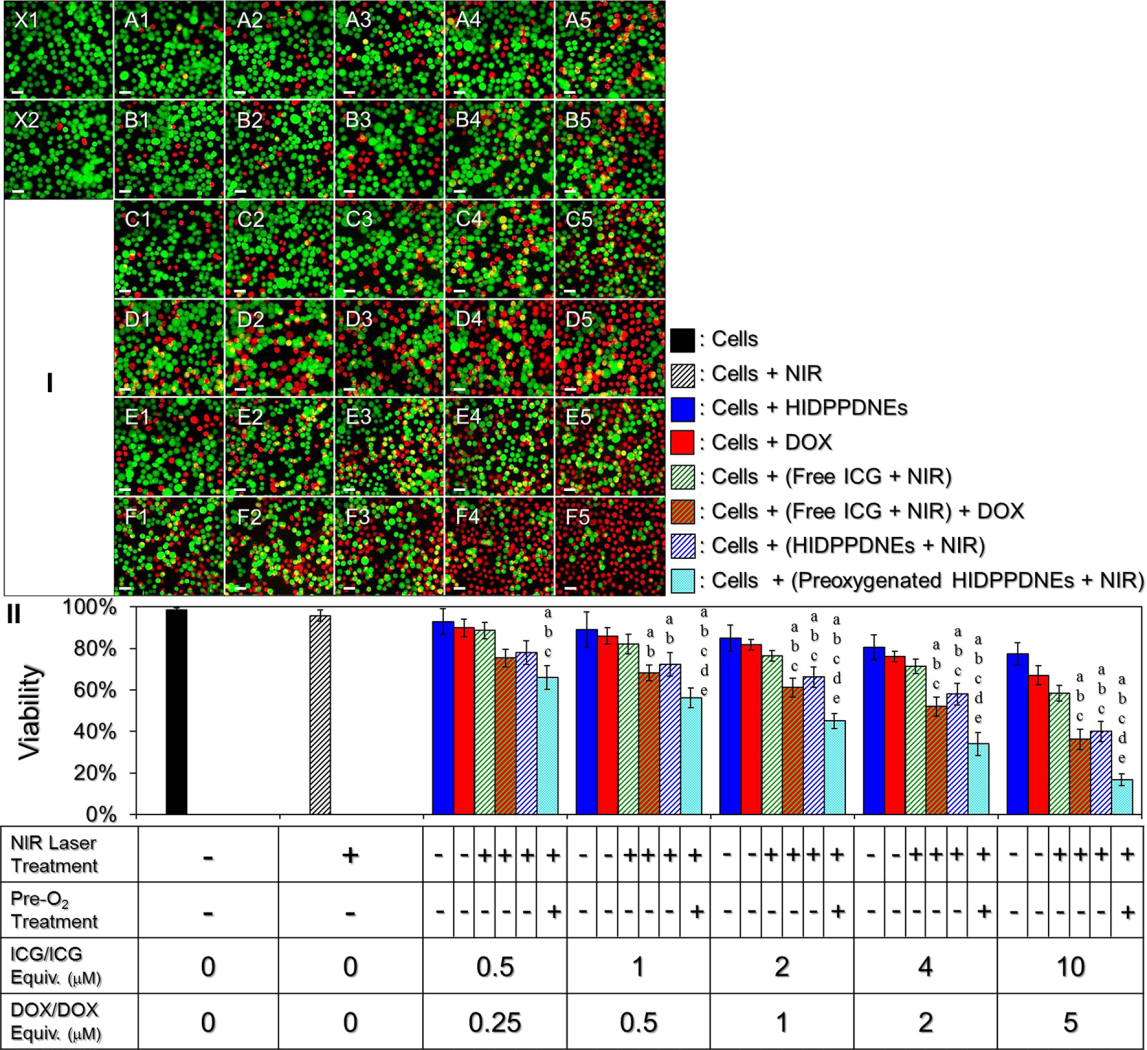
Cytotoxicity of the HIDPPDNEs to HER2(+) breast cancer cells in vitro. **I** Photomicrographic images of MDA-MB-453 cells under various treatments. Rows *A* and *B* represent the groups that cells were co-cultured with the HIDPPDNEs (*A*) or free DOX (*B*) for continuous 24 h in which the concentrations of DOX were set at 0.25 (*A1* and *B1*), 0.5 (*A2* and *B2*), 1 (*A3* and *B3*), 2 (*A4* and *B4*), and 5 (*A5* and *B5*) μM. Rows *C* and *D* represent the groups that cells were pre-co-cultured with 0.5 (*C1* and *D1*), 1 (*C2* and *D2*), 2 (*C3* and *D3*), 4 (*C4* and *D4*), and 10 (*C5* and *D5*) μM of free ICG for 4 h, then treated with NIR irradiation for 5 min followed by incubation at 37 °C in the absence (Row *C*) or presence of 0.25- (*D1*), 0.5- (*D2*), 1- (*D3*), 2- (*D4*), and 5- (*D5*) μM DOX for 24 h. Rows *E* and *F* represent the groups that cells were pre-co-cultured with HIDPPDNEs (*E*) or preoxygenated HIDPPDNEs (*F*) for 4 h in which the concentrations of ICG and DOX contained (ICG/DOX) were set at 0.5/0.25 (*E1* and *F1*), 1/0.5 (*E2* and *F2*), 2/1 (*E3* and *F3*), 4/2 (*E4* and *F4*), and 10/5 (*E5* and *F5*) μM, then treated with NIR irradiation for 5 min followed by incubation at 37 °C for 24 h. *X1* denotes the cells with neither drug (ICG and/or DOX) nor NIR exposure. *X2* represents the cells treated with NIR irradiation for 5 min followed by incubation at 37 °C for 24 h. The cells in row *C*, *D*, *E*, and *F* were washed twice with PBS before NIR exposure. The green and red cells stained by calcein-AM and PI represent live and dead cells, respectively. All images were photographed using a fluorescence microscope at ×200 magnification. *Scale bar* 30 μm. **II** Quantitative analyses of the viabilities of MDA-MB-453 cells after treatment with DOX, free ICG, or HIDPPDNEs in different conditions as indicated in the *X-axis*. Values are mean ± s.d. (n = 3). ^a^ *P* < 0.05 as compared to the HIDPPDNE-treated group without NIR exposure. ^b^ *P* < 0.05 as compared to the group with equal concentration of free DOX. ^c^ *P* < 0.05 as compared to the group with equal concentration of free ICG and NIR irradiation. ^d^ *P* < 0.05 as compared to the group with equal dose of free ICG and NIR irradiation followed by incubation with DOX. ^e^ *P* < 0.05 as compared to the group with equal dose of HIDPPDNEs and NIR irradiation

Figure 11 shows the viabilities of MDA-MB-453 cells after treatments with various doses of ICG, DOX, or HIDPPDNEs with and without preoxygenated treatment and/or NIR laser irradiation (808 nm, 6 W/cm^2^). The concentrations of free ICG and DOX examined in the cytotoxicity experiments are corresponding to the dosages provided by the HIDPPDNEs. Based on the hemocytometric analyses (Fig. 11II), the viability of the cells treated with NIR alone (Fig. 11I, X2) was 95.8%, indicating that the slight temperature increase due to NIR laser irradiation (Fig. 8) was nontoxic. On the other hand, a dose-dependent increased cytotoxicity was obtained in each conditional group (Fig. 11I; row A–F), and the results show that the cells treated with the HIDPPDNEs and NIR irradiation (Fig. 11I, row E) underwent higher mortality rate as compared to the cells treated with the HIDPPDNEs without NIR exposure (Fig. 11I, row A), those treated with DOX alone (Fig. 11I, row B), and those treated with free ICG and NIR irradiation (Fig. 11I, row C) (*P* < 0.05 for all comparisons when the dose of HIDPPDNE was ≥2-μM ICG/1-μM DOX equivalent concentration), whereas the level of cell death was similar with that obtained from the cells with free ICG/DOX-mediated photochemotherapy (Fig. 11I; row D). These outcomes demonstrate that the HIDPPDNEs are certainly effective on the cancer cell eradiation upon NIR irradiation (808 nm; 6 W/cm^2^), but less toxic in the absence of NIR exposure. Moreover, we surprisingly found that the preoxygenated HIDPPDNEs with NIR irradiation (Fig. 11I, row F) were able to provide the highest efficacy of cell destruction as compared with other settings that the viabilities of 66, 56.2, 45, 34, and 16.8% were obtained for the groups with 0.5-/0.25-, 1-/0.5-, 2-/1-, 4-/2-, and 10-/5-μM of ICG/DOX, respectively, and those viabilities are 1.2-, 1.3 (*P* < 0.05)-, 1.5 (*P* < 0.05)-, 1.8 (*P* < 0.05)-, and 2.5 (*P* < 0.05)-fold lower than the values obtained from the cells with equal amount of HIDPPDNEs without preoxygenated treatment.

Based on the results shown in Fig. 9a that the treatment of preoxygenation may reduce production of singlet oxygen yielded from the HIDPPDNEs, one may question that how the efficacy of cell eradication in the group with preoxygenated HIDPPDNEs was higher? We speculate that it is because instead of the protocol operated for examining the photodynamic effect of the HIDPPDNEs that the nanodroplets were treated with the NIR laser immediately after placed in the wells, in the cytotoxicity experiment, the unbound HIDPPDNEs, no matter with or without preoxygenation, were removed after 4-h co-culture with the cells and thus led to a decrease of oxygen amount in the culture system. Furthermore, an O_2_–CO_2_ gas exchange may occur in the HIDPPDNEs when they were placed in the 5% CO_2_-enriched incubator since the capability of PFC in CO_2_ absorption is much higher than that in O_2_ absorption [22]. Taken all together, we therefore reason that the preoxygenated HIDPPDNEs may be able to preclude the hyperoxia limitation for the singlet oxygen generation and thereby provide enhanced photodynamic effect due to higher oxygen payload as compared to the HIDPPDNEs without preoxygenation, leading to a higher anticancer efficiency as shown in Fig. 11. Considering the effects of nanoparticle diffusion, enzymatic attack, and multiple drug clean-up mechanisms carried out by such as reticuloendothelial system and transcapillary filtration in physiology, quite a few nanodroplets are predicted to be off-target and/or eliminated while utilized in vivo and thus the scenario of HIDPPDNE-induced hyperoxia at the tumor site may not occur in the practice. Therefore, in terms of the application of HIDPPDNE in the clinic, it is recommended to process the preoxygenation for the HIDPPDNEs to increase the quantity of oxygen loaded in each nanodroplet before use and thereby enhance the anticancer efficacy as illustrated in Fig. 11.

To minimize the potential chemotherapy-induced side effects, in this study, the HIDPPDNEs were used with up to 5 μM of DOX and that is lower than the dose typically used in the clinic (≥10 μM) [46]. However, a robust cytotoxicity of the HIDPPDNEs upon NIR irradiation was still obtained in each setting and the resulting mortality was even higher than that caused by using twice amount of encapsulated DOX alone (Fig. 11II), indicating that the phototherapy indeed played a crucial role in the HIDPPDNE-mediated cancer treatment. The importance of phototherapy in such combined therapeutics can also be identified through comparing the viability of cells treated by free DOX alone (Fig. 11I; row B) to that treated with both free ICG and DOX (Fig. 11I; row D). In this study, although the effect of photochemotherapy generated from the HIDPPDNEs in vitro can be achieved by using free ICG and DOX individually (Fig. 11I; row D versus E), it is predictable that the latter may not be an appropriate approach for use in the clinic due to a variety of defects such as lack of target-ability, insufficient stability, and rapid plasma clearance for the free ICG as described above. With merits of improved stability, resistibility to macrophage clearance, and HER2 binding specificity, the HIDPPDNEs are considered to be a more beneficial photosensitizer than free ICG in terms of use for phototherapy. Moreover, the HIDPPDNEs with preoxygenation may further enhance the efficacy of cancer cell eradication that is highly advantageous for use in the clinic.

## Conclusions

In this study, we have successfully fabricated HIDPPDNEs for targeted photochemotherapy of HER2(+) breast cancer cells. We not only investigated their physicochemical properties and functionalities, but also examined the availability of the developed nanodroplets for use in the cancer cell eradication in vitro. In addition to the aforementioned advantages of the HIDPPDNEs, the constituent PEI oriented toward the external aqueous phase may allow the nanodroplets efficiently escape the endosome/lysosome due to the proton sponge effect provided [47], and therefore the intracellular delivery rate of the loaded drugs can be facilitated and lead to an enhanced therapeutic effect accordingly. Moreover, it has been known that the nonionic PEO/PPO block copolymer, such as Pluronic F68 used in this study, may reduce P-glycoprotein activity of the drug-resistant cells by decreasing their ATP production [48], and that may diminish the level of medicinal resistance in the HER2-overexpressing breast cancer cells accordingly. Taken all together, we anticipate that the HIDPPDNEs may be able to provide an improved cancer therapy in vivo as compared to that treated by free ICG and/or DOX alone. However, further studies are certainly needed and efforts are currently in progress.

## Additional file

**Additional file 1: Figure S1.** Representative graphs of size distribution for IDPDNEs (A), IDPPDNEs (B), and HIDPPDNEs (C) measured by DLS technique. **Figure S2.** Representative graphs of zeta potential (/surface charge) for IDPDNEs (A), IDPPDNEs (B), and HIDPPDNEs (C) measured by DLS technique.

## Abbreviations

HER2: human epidermal growth factor receptor 2
DOX: doxorubicin
NIR: near infrared
ROS: reactive oxygen species
PTT: photothermal therapy
PDT: photodynamic therapy
ICG: indocyanine green
PEI: polyethyleneimine
PFC: perfluorocarbon
HIDPPDNEs: anti-HER2 ICG–DOX-loaded PEI-coated PFC double nanoemulsions
PEO: poly(ethy1ene oxide)
PPO: poly(propy1ene oxide)
DMAP: dimethylaminopyridine
TEA: triethylamine
CT-PF68: carboxyl-terminated Pluronic F68
IDPDNEs: ICG–DOX-loaded PFC double nanoemulsions
PFOB: perfluorooctyl bromide
IDPPDNEs: ICG–DOX-loaded PEI-coated PFC double nanoemulsions
IgG: immunoglobulin G
F-IgG-SAb: fluorescent anti-mouse IgG secondary antibody
DLS: dynamic light scattering
*k*_d_: degradation rate coefficient
RFUs: relative fluorescence units
s.d.: standard deviation
