# Supplementary material for: Synthesis, characterization, and biological verification of anti-HER2 indocyanine green–doxorubicin-loaded polyethyleneimine-coated perfluorocarbon double nanoemulsions for targeted photochemotherapy of breast cancer cells
Source: J Nanobiotechnology. 2017 May 18;15:41. doi: 10.1186/s12951-017-0274-5 (PMC5437512; doi:10.1186/s12951-017-0274-5)
Supplement: Supplementary file 1 — Additional file 1: Figure S1. Representative graphs of size distribution for IDPDNEs (A), IDPPDNEs (B), and HIDPPDNEs (C) measured by DLS technique. Figure S2. Representative graphs of zeta potential (/surface charge) for IDPDNEs (A), IDPPDNEs (B), and HIDPPDNEs (C) measured by DLS technique. [file 12951_2017_274_MOESM1_ESM.docx]

Supplementary Materials
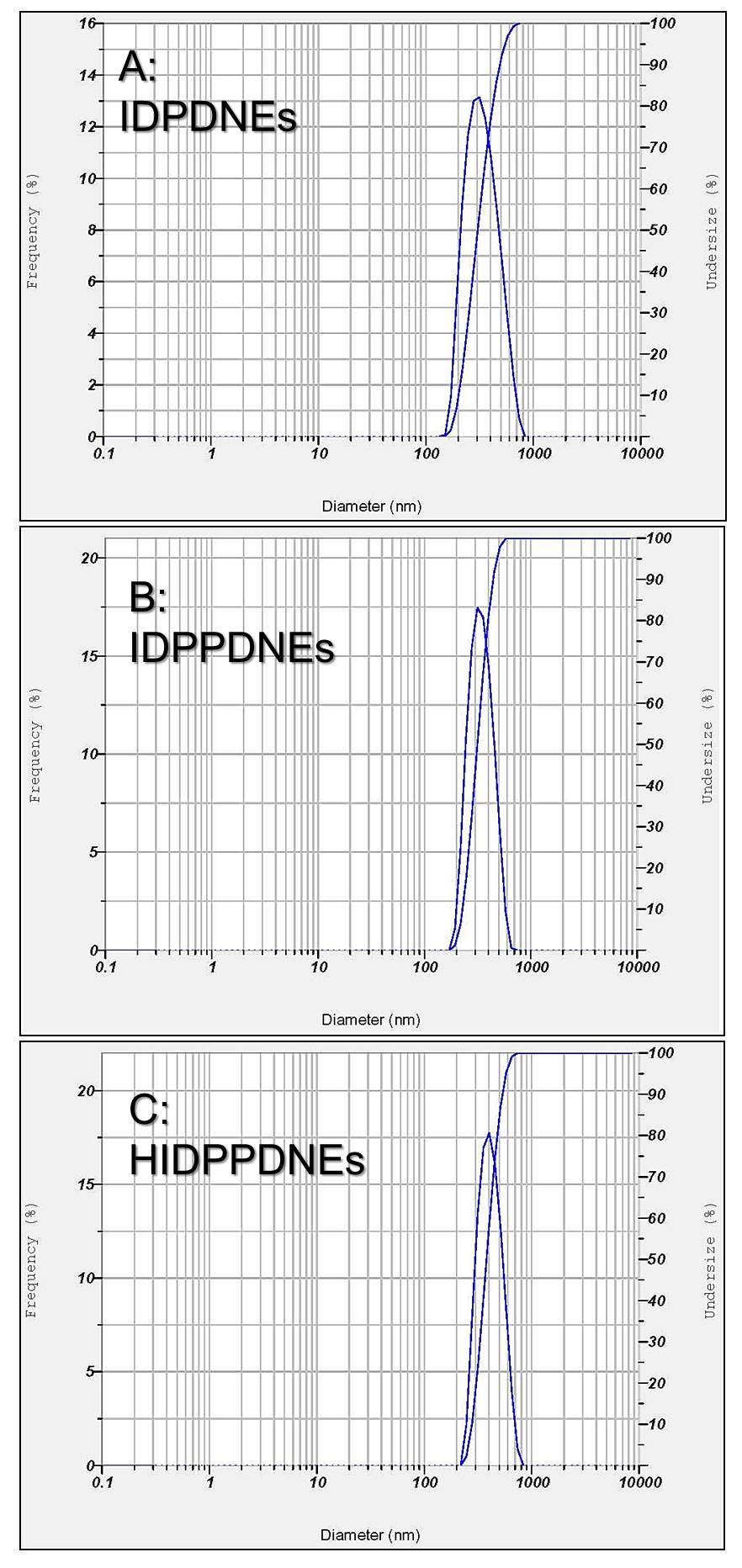


**Fig. S1** Representative graphs of size distribution for IDPDNEs (A), IDPPDNEs (B), and HIDPPDNEs (C) measured by DLS technique.


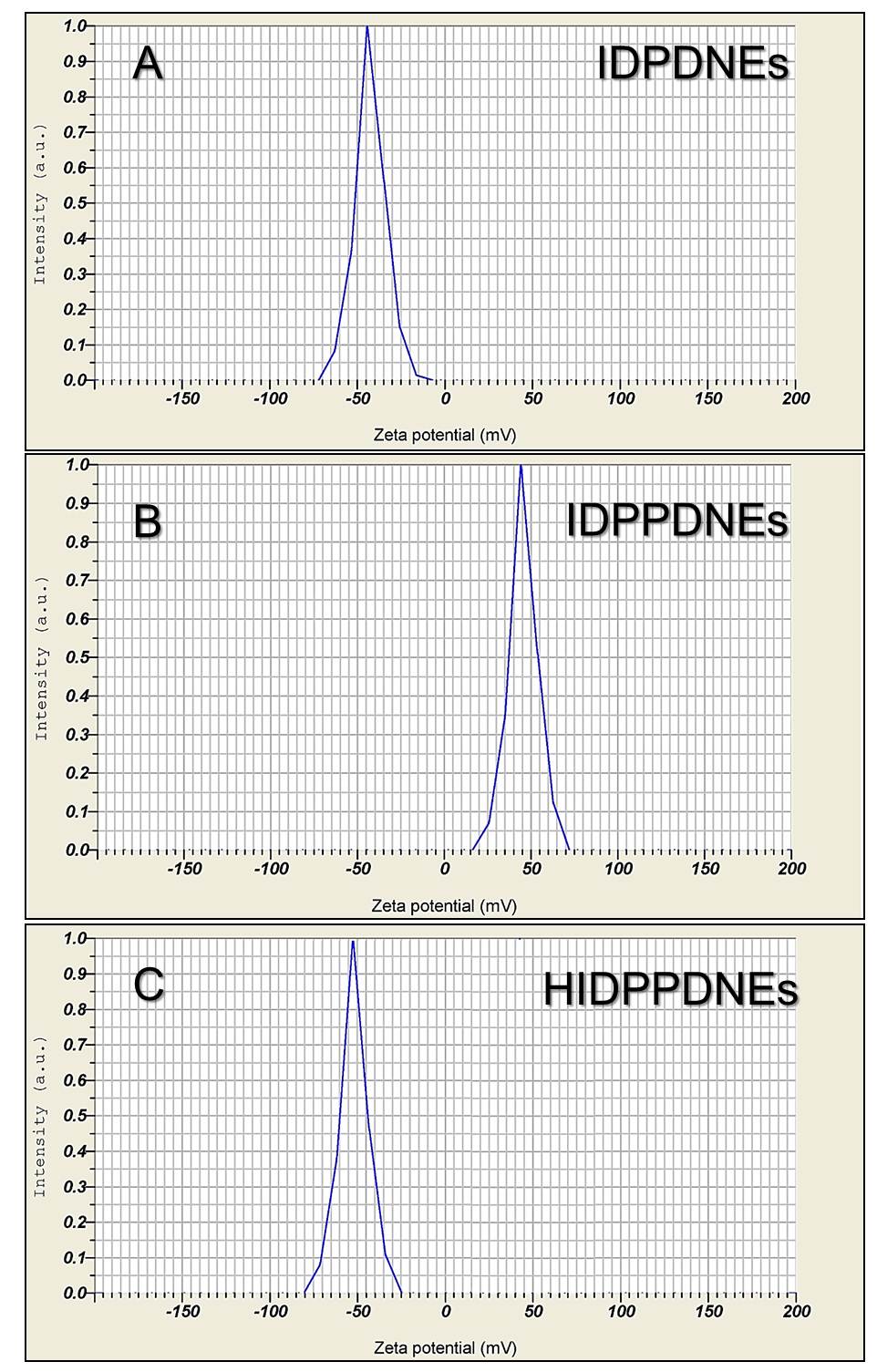


**Fig. S2** Representative graphs of zeta potential (/surface charge) for IDPDNEs (A), IDPPDNEs (B), and HIDPPDNEs (C) measured by DLS technique.
